# Alterations of Neuromuscular Function after the World's Most Challenging Mountain Ultra-Marathon

**DOI:** 10.1371/journal.pone.0065596

**Published:** 2013-06-26

**Authors:** Jonas Saugy, Nicolas Place, Guillaume Y. Millet, Francis Degache, Federico Schena, Grégoire P. Millet

**Affiliations:** 1 Institute of Sport Sciences, Faculty of Biology and Medicine, University of Lausanne, Lausanne, Switzerland; 2 Institute of Movement Sciences and Sports Medicine, University of Geneva, Geneva, Switzerland; 3 Universite de Lyon, Saint-Etienne, France and Exercise Physiology Laboratory, Jean Monnet University, Saint-Etienne, France; 4 Faculty of Motor Sciences, University of Verona, Verona, Italy; The University of Queensland, Australia

## Abstract

We investigated the physiological consequences of the most challenging mountain ultra-marathon (MUM) in the world: a 330-km trail run with 24000 m of positive and negative elevation change. Neuromuscular fatigue (NMF) was assessed before (Pre-), during (Mid-) and after (Post-) the MUM in experienced ultra-marathon runners (n = 15; finish time  = 122.43 hours ±17.21 hours) and in Pre- and Post- in a control group with a similar level of sleep deprivation (n = 8). Blood markers of muscle inflammation and damage were analyzed at Pre- and Post-. Mean ± SD maximal voluntary contraction force declined significantly at Mid- (−13±17% and −10±16%, P<0.05 for knee extensor, KE, and plantar flexor muscles, PF, respectively), and further decreased at Post- (−24±13% and −26±19%, P<0.01) with alteration of the central activation ratio (−24±24% and −28±34% between Pre- and Post-, P<0.05) in runners whereas these parameters did not change in the control group. Peripheral NMF markers such as 100 Hz doublet (KE: −18±18% and PF: −20±15%, P<0.01) and peak twitch (KE: −33±12%, P<0.001 and PF: −19±14%, P<0.01) were also altered in runners but not in controls. Post-MUM blood concentrations of creatine kinase (3719±3045 Ul·^1^), lactate dehydrogenase (1145±511 UI·L^−1^), C-Reactive Protein (13.1±7.5 mg·L^−1^) and myoglobin (449.3±338.2 µg·L^−1^) were higher (P<0.001) than at Pre- in runners but not in controls. Our findings revealed less neuromuscular fatigue, muscle damage and inflammation than in shorter MUMs. In conclusion, paradoxically, such extreme exercise seems to induce a relative muscle preservation process due likely to a protective anticipatory pacing strategy during the first half of MUM and sleep deprivation in the second half.

## Introduction

Mountain ultra-endurance running has experienced considerable growth in recent years. These events consist of running/walking on mountain trails with positive and negative slopes over a distance longer than the traditional marathon. These extreme events are an opportunity to investigate the physiological responses of the human body when pushed to its limits [1]. Some previous studies have already assessed the acute consequences of these mountain ultra-marathons (MUM) on inflammation, neuromuscular or cardiac fatigue or hemolysis [2]–[5] but none of them have assessed the consequences of an event longer than 50 h. More precisely, neuromuscular fatigue has been assessed after 5 to 37 h prolonged running exercises and the general observation was the presence of large central fatigue [3], [4], [6] with some slight differences depending of the muscle group tested, usually knee extensors (KE) *vs.* plantar flexors (PF). Moreover, peripheral fatigue, such as low-frequency fatigue, has also been reported after a MUM [3]. A plateau in the maximal voluntary contraction (MVC) force loss (index of global fatigue) has been suggested [7]; it appears that after approximately 30–36 h of running, MVC force loss does not increase further. However, to our knowledge, there are no data available on MUM longer than 37 h to confirm the presence of this plateau.

Information about inflammation and muscle damage have already been collected after a MUM [2], [3], [5] and revealed large increases of myoglobin, C-reactive protein, lactate dehydrogenase (LDH) and creatine kinase (CK) concentrations. For example, CK activities have been reported to be ∼13,600 UI·L^−1^ (mean value) after the Ultra-trail du Mont-Blanc (UTMB). Such levels are similar to those measured in patients with rhabdomyolysis [8] and confirm the extreme solicitation of the organism.

Analyzing MUM longer than 100 hours involves sleep deprivation, which is not an issue in shorter events. This factor has never been assessed in previous MUM studies lasting less than 50 h. Little is known about the effects of sleep deprivation on neuromuscular fatigue and muscle damage. It has been reported that 24 h of sleep deprivation did not affect weightlifting performances [9]. Following 30-h sleep deprivation, isokinetic knee flexion peak torque decreased but no effects were detected on fatigue index [10]. Electrically evoked force or maximal voluntary strength did not change during either isometric or isokinetic contractions of the upper or lower limbs during 60 h of sleep deprivation [11], [12]. These findings suggest that sleep deprivation *per se* has little effect on the neuromuscular function.

Analyzing MUM longer than 100 hours also allows making a mid-run measurement, which is difficult in shorter duration MUMs. Effectively, with such a long exercise time, a few minutes lost for the study won't have any effect on the final performance. Finally, the comparison of runners and control subjects has never been performed on MUM studies.

The aim of the present study was to investigate the etiology of neuromuscular fatigue, muscle damage and inflammation in the most extreme (duration: 80–150 h) MUM in the world. By using data recorded prior to, during and after the event on both runners and control subjects, we aimed at characterizing the specificities of such MUM and better describe the underlying mechanisms of extreme fatigue.

## Methods

### Experimental design

**Figure 1:**
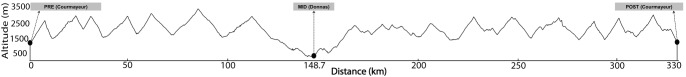
Altitude profile of the entire run with the three session test locations and the distance scale in km.

The race supporting this study was the *Tor des Géants* (TdG) 2011. It consisted of running/walking ∼330 km around the province of Val d'Aoste in Italy with a total positive and negative elevation change of ∼24000 m (Figure 1). There were 471 starters and 301 finishers (64%) in 2011. This race is considered as the world's most challenging single-stage MUM. The maximum and minimum altitudes are 3300 m and 322 m, respectively. There are 20 passes over 2000 m. The maximum time allowed for completion of the race is 150 h and the current record is 76 h. The distance is divided into seven parts interspersed by six aid-stations where sleeping is allowed. However, the participants do not have any compulsory stops and therefore can pace themselves and manage their stops as they wish. Since the recovery time (e.g. nutrition, hydration, sleep) is not subtracted from the race time, the influence of pacing and sleep deprivation is paramount. This race is therefore different to other mountainous ultra-trails of shorter distances (e.g. Ultra-trail du Mont-Blanc, UTMB, 166 km [3]) or road ultra-marathons over longer distance but with several stages (e.g. Trans Europe Foot Race, 4,487 km in 64 stages from South Italy to North Cape, Norway in 2009, [13]) where sleep management is of less importance.

The study was approved by the institutional ethics committee of the University of Verona, Italy (Department of Neurological, Neuropsychological, Morphological and Motor Sciences). All subjects provided written, voluntary, informed consent before participation. The experiment was conducted according to the Declaration of Helsinki. Most of the subjects were familiar with testing procedures, having previously participated in similar experiments [3], [6].

### Participants

Twenty-five male runners took part in the present study. Participants were tested three times: before the run (Courmayeur, Italy, altitude 1224 m, km 0, Pre-); during the run (Donnas, Italy, altitude 322 m, km 148.7, Mid-); and approximately 30 minutes after the run (Courmayeur, Italy, altitude 1224 m, km 330, Post-).

Of the 25 initially engaged participants, 15 (*i.e.* 60%) completed the TdG and 9 took part in the three testing sessions (*i.e.,* Pre-, Mid- and Post-). The finishers/starters ratio for our subjects was similar to the overall ratio for this race (64%). All subjects were experienced in ultra-marathons/trails (Table 1). Three subgroups of runners (Pre-Mid-Post, n = 9; Pre-Mid, n = 15; Pre-Post, n = 15) were considered in the present study (see statistical analysis for more details). There was no significant difference between the three groups (n = 9 and n = 15) for any characteristics (Table 1). Therefore, the group “Pre-Mid-Post” (n = 9) was considered as representative and was renamed “TOR”.

**Table 1:** Main characteristics of the different groups.

Group	N	Age (yr)	Height (cm)	Mass (kg)	Body fat (%)	Training (h·wk^−1^)	Year of expe rience in trail running (yr)	Sleep at Mid (h)	Sleep at Post (h)
**Total runners**	25	45.4±10.3	174±5	69.4±6.1	18.4±2.9	8.2±5.1	7.7±5.3	-	-
**“Pre-Mid-Post” (TOR)**	9	41.6±13.1	174±6	67.3±6.4	17.8±3.3	7.2±4.1	7.8±6.9	1.2±1.6	8.6±5.2
**“Pre-Mid”**	15	45.1±11.9	174±6	71.0±6.2	17.5±3.3	7.1±4.4	8.1±5.5	1.9±2.1	9.9±5.4
**“Pre-Post”**	15	44.7±11.3	174±5	67.9±5.8	18.4±3.2	7.8±5.2	7.4±5.5	1.4±1.8	9.1±4.8
**Control (CON)**	8	29.3±8.1#	174±6	70.9±9.3	20.1±6.1	2.7±2.5	0.8±1.2	1.2±1.8	12.3±5.4

Data are mean ± SD. Training volume was considered for the past trail running season. Mid-: during the run at Donnas, km 148.7. Post-: about 30 minutes after the race, at Courmayeur. Sleep represents the sleeping time accumulated at Mid- and Post-. #: P<0.001 for differences between control and other groups.

In parallel, a control group (CON, n = 8) participated in this study, and was tested at Pre- and Post-. Table 1 shows that TOR and CON were similar except for age and experience in trail running. The timing for the Pre- and Post- measurements was strictly similar in TOR and CON. This control group was constituted of investigators. Of importance is that both groups were constrained by the same level of sleep deprivation (Table 1).

### Force assessment

MVC force loss of KE and PF was evaluated to provide an index of global fatigue [14]. The voluntary activation ratio of KE and PF was assessed using superimposed high-frequency (100 Hz) doublet to detect central fatigue. Finally, evoked stimulations were delivered to the relaxed muscle in a potentiated state to determine the extent and type of peripheral fatigue.

The protocol was identical for the three testing sessions, and was conducted as follows:

1. One MVC (duration ∼4 s).
2. One or two MVCs with a superimposed 100 Hz doublet, followed by a 100 Hz doublet (∼2 s after the MVC), a 10 Hz doublet and a single twitch (∼2–3 s between each stimulation). A second MVC was performed if a plateau was not observed in the first one.

This sequence was conducted twice at Pre- and Post- and only one time at Mid- since the time was limited for the runners during the race. At Pre-, the first step consisted of determining the supramaximal stimulation intensity, which was used for all sessions. In Pre-, subjects performed 8–10 contractions on each ergometer at intensities ranging from 20–80% of the estimated MVC as a warm-up. No warm-up was performed for Mid- or Post-.

For the KE testing, subjects were seated in an isometric ergometer comprised of a custom-built chair equipped with a strain gauge (STS 250 kg, sensitivity 2.0005 mV·V^−1^ and 0.0017 V·N^−1^, SWJ, China). Subjects were seated with a knee angle of 90° and a trunk-thigh angle of 100° (180° =  neutral position). The strain gauge was attached to the chair on one end and securely strapped to the ankle with a custom made mould. Two crossover shoulder harnesses and a belt limited extraneous movement of the upper body across the lower abdomen. Subjects were instructed to cross their arms on the trunk during the MVC. During the MVCs, subjects were strongly encouraged.

For the PF testing, another specific custom-built ergometer was used. The forefoot was strapped on this ergometer and the knee was blocked to limit heel lift during the MVC. Arms were crossed on the trunk. The hip, knee and ankle angles were set at 90 degrees. Subjects were sitting on a massage table vertically adjustable. Mechanical torque of the PF muscles was obtained with an instrumental pedal equipped with a strain gauge sensor (Vishay Micro Measure, Raleigh, NC) set in bridge of *Wheatstone* (set of two perpendicular gauges). The pedal's body was made of aluminum (Fortal 7075-T6, Grenoble, France) with an elastic limit over 500 MPa. The field of the torque measured and tested extended to 30 kg·m. The lower section of the pedal body was 60 mm wide and 16 mm thick, with a constraint of 120 MPa. To limit the contribution of muscle groups other than PF and to optimize force recordings, the upper leg was clamped down to the pedal just proximal to the knee. Mechanical data were recorded at 1 kHz using an AD conversion system (MP150; Biopac system, Goleta, CA). The right leg was investigated for all subjects in both muscle groups. It is important to note, that the right leg was the dominant one for all subjects. For both KE and PF testing, the settings of the ergometer were noted to standardize the position of every subject.

### Electrical stimulation

Most of the subjects (75%) had already experienced electrical stimulation of femoral and tibial nerves [3], [6]; thus no familiarization session was performed. Transcutaneous stimulations were induced by a high-tension, constant current stimulator (maximal voltage of 400 V, DS7AH, Digitimer, Hertfordshire, UK). The doublets were induced by a Train/Delay Generator (Model DG2A, Digitimer).

For KE, the cathode (5-cm diameter, Dermatrode, American Imex, Irvine, CA) and the anode (5×10 cm, Medicompex SA, Ecublens, Switzerland) were placed over the femoral nerve at the femoral triangle level beneath the inguinal ligament and on the lower part of the gluteal fold opposite to the cathode, respectively. For the PF, the anode (10×5 cm, rectangular self-adhesive stimulation electrode, Medicompex SA) was located on the inferior base of the quadriceps muscle and the cathode (EMG electrode, 10 mm diameter, Kendall Meditrace 100, Tyco, Canada) was located on the tibial nerve in the popliteal cavity.

The maximal stimulation intensity was measured during the first testing session by progressively increasing the intensity until a plateau was observed for the mechanical (peak twitch) and electrical (M-wave amplitude) responses. This stimulation intensity was then increased by 50% to obtain supramaximal intensity.

### Electromyographic recordings

The EMG signals of the right *vastus lateralis* (VL) and *soleus* (SOL) were recorded using bipolar silver chloride surface electrodes of 10-mm diameter (Kendall Meditrace 100) during the MVC and electrical stimulation. These muscles were chosen as VL has been proposed as a surrogate for the quadriceps muscle [15] and SOL is the main muscle contributing to plantar flexor force at a knee angle of 90° [16]. The recording electrodes were taped on the skin over the muscle belly following SENIAM recommendations [17], with an inter electrode distance of 20 mm [18]. The position of the electrodes was marked on the skin so that they could be fixed on the same place for the two other sessions. The reference electrode was attached on the patella (for both VL and SOL EMG). Low impedance (<10 kΩ) at the skin-electrode contact was obtained by shaving,abrading the skin with an abrasive sponge and cleaning with alcohol. EMG data were recorded with Biopac system (MP150, Biopac System, Goleta, CA) and amplified (gain = 1000) with a bandwidth frequency ranging from 10 to 500 Hz, digitized at a sampling frequency of 2 kHz, and recorded by the AD conversion system (common mode rejection ratio: 90 dB, input impedance: 100 MΩ; gain: 1000). Isometric force and EMG data were stored and analyzed offline with commercially available software (Acqknowledge 4.1 software, Biopac System, Goleta, CA).

### Blood samples

Peripheral venous blood was collected at Pre- and Post- by peripheral venipuncture into siliconized vacuum tubes containing either K2 EDTA (Becton-Dickinson, Oxford, UK) for blood count analysis (Advia 2120, Siemens, Germany) or lithium heparin for clinical chemistry testing (Modular Analytics, Roche, Switzerland). The samples were stored in a refrigerated bag and transported to the reference laboratory for analysis. The quality of the results was validated by regular internal quality control procedures and participation in an External Quality Assessment Scheme. For further details, see [19], [20].

#### General fatigue and peripheral pain

Subjects were requested to quantify the levels of general fatigue and subjective pain (e.g. muscle soreness, joint pain) in three anatomical areas (foot-ankle; leg-knee; thigh-hip) and their digestive feelings by using a visual analog scale (VAS) with a 100-mm horizontal line with “no fatigue/no pain” on one end (0 mm) and “extremely fatigued/painful” on the other (100 mm). Subjects were asked to mark their pain level on the VAS under supervision of an examiner.

### Mechanical responses

Peak force measured during the highest MVC for each session was considered as the MVC value. The amplitudes of the potentiated low- (10 Hz, PS10) and high-frequency (100 Hz, PS100) doublets, potentiated twitch (Pt) and superimposed high-frequency doublet and the ratio of high- to low-frequency doublet peak force (PS10/100) were analyzed for the KE and the PF. For these parameters, the values obtained during or after the highest MVC were considered for each muscle group. The PS10/100 ratio was used to assess the development of low frequency fatigue (LFF) [21].

### MVC force and central activation

The Central Activation Ratio (CAR) was was calculated as follows [22]:
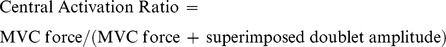



### EMG

The M-wave peak-to-peak duration and amplitude were measured from VL and SOL muscles. They were analyzed from the single potentiated twitch evoked on the relaxed muscle. The EMG Root Mean Square (RMS) values for VL and SOL muscles at peak torque level were calculated during the highest MVC trial over a 500 ms period (250 ms period either side of the peak torque).

### Statisticical analysis

Data were screened for normality of distribution and homogeneity of variance using a Shapiro-Wilk normality test and the Barlett's test, respectively.

First, we tested differences across the testing sessions for the three different experimental groups (TOR, n = 9; Pre-Mid, n = 15; Pre-Post, n = 15). When conditions of analysis of variance (ANOVA) application were met, each variable was compared between the different times of measurements (Pre-Mid-Post, n = 9; Pre-Mid, n = 15; Pre-Post, n = 15) using a repeated measures ANOVA for the experimental sub-groups. Bonferroni corrections were applied to determine between-means differences if the ANOVA revealed a significant main effect. When conditions of application for parametric repeated-measures ANOVA were not met, Friedman ANOVA was used.

Second, we examined the TOR vs CON differences: a repeated measures ANOVA was used to identify differences by examination of the group (TOR vs CON) x time (Pre- vs. Post-) interaction, complemented by a Bonferroni post-hoc test. When the conditions of application were not met, a Mann-Whitney rank-sum test was used.

Third, for comparison of the percent decreases in neuromuscular parameters between TdG and a previous study with the same experimental method (i.e. UTMB [3]), unpaired t-tests were used.

Pearson correlation coefficients were calculated between Pre- to Post-MUM changes in neuromuscular and blood parameters.

For all statistical analyses, a P value of 0.05 was accepted as the level of significance. All data presented as mean values ± SD in the text and tables and as mean values ± SE in figures.

## Results

### Considerations about groups and subgroups

There was no significant difference between groups (TOR, Pre-Post and Pre-Mid) in terms of age, height, weight and running time. Furthermore, for all subsequent analyses and discussion, only the TOR group has been used. However, we did report the changes for the other two sub-groups in the figures to show that the TOR group was representative of the other groups, as done in a previous study. [3]

### Performance and muscle fatigue

The average finishing time of our subjects was 122.43 hours ±17.21 hours, their final rank being from 8^th^ to 244^th^ position out of 301 finishers.

**Figure 2:**
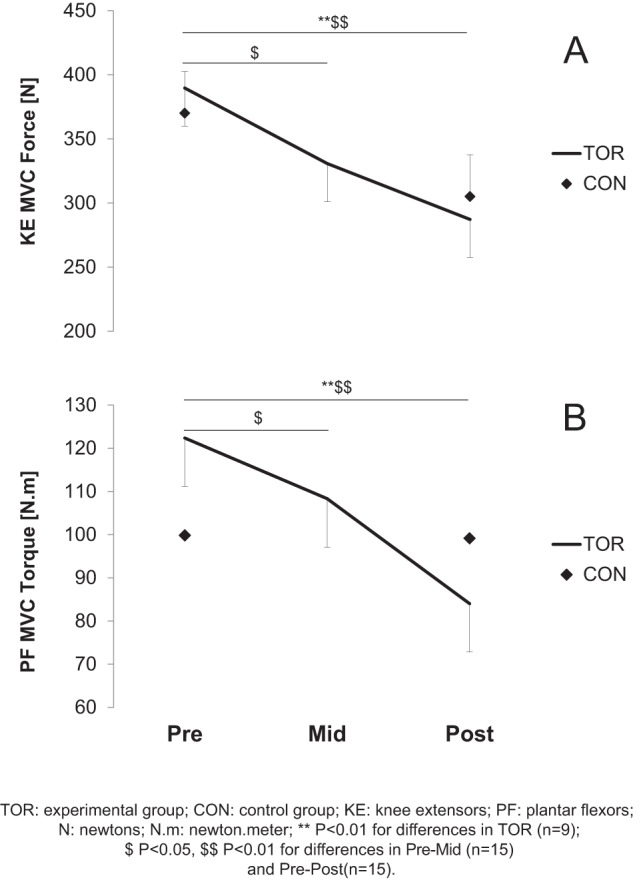
Maximal voluntary contraction (MVC) prior to (Pre-), in the middle of (Mid-) and after (Post-) the race for knee extensors (KE, panel A) and plantar flexors (PF, panel B). A two-way repeated measure ANOVA was used complemented by a Bonferroni post-hoc test. Values are mean ± SE.

MVC declined significantly (Figure 2) at Mid- (−12.6±17.4% and −9.7±16.4% for KE and PF, respectively; P<0.05), and further decreased at Post- (−23.9±13.1%; P<0.01 and −26.4±19.1%, respectively; P<0.001). For the control group, MVC was not significantly reduced between Pre- and Post- both for KE (−16.5±15.1%) and PF (−1.6±12.9%).

### Central fatigue

CAR decreased significantly for both KE and PF (−22.1±16.2% and −29.2±24.5% for KE and PF respectively) muscles in TOR but no change was observed in CON (Table 2).

**Table 2:** Central activation ratio (CAR, %) for the knee extensors (KE) and the plantar flexors (PF) prior to (Pre-), in the middle of (Mid-) and after (Post-) the race in runners (TOR, n = 9) and control group (CON, n = 8).

CAR (%)	KE			PF		
	Pre	Mid	Post	Pre	Mid	Post
**TOR**	91.2±5.9	87.5±8.7	71.1±26.5*	89.0±10.3	87.3±13.7	63.0±38.7*
**CON**	88.5±6.8	-	82.3±10.1	85.7±17.2	-	83.8±15.1

Values are given as mean ± SD. *: P<0.05 for differences in TOR between Pre- and Post-.

### Peripheral fatigue

**Figure 3:**
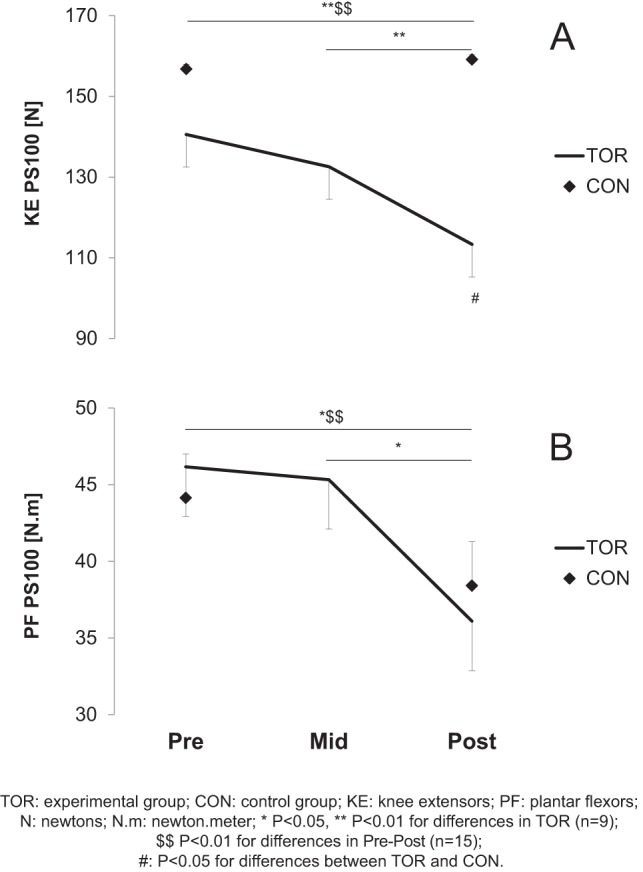
Amplitude of the potentiated doublet (PS100) prior to (Pre-), in the middle of (Mid-) and after (Post-) the race for knee extensors (KE, panel A) and plantar flexors (PF, panel B). A two-way repeated measure ANOVA was used complemented by a Bonferroni post-hoc test. Values are mean ± SE.

**Figure 4:**
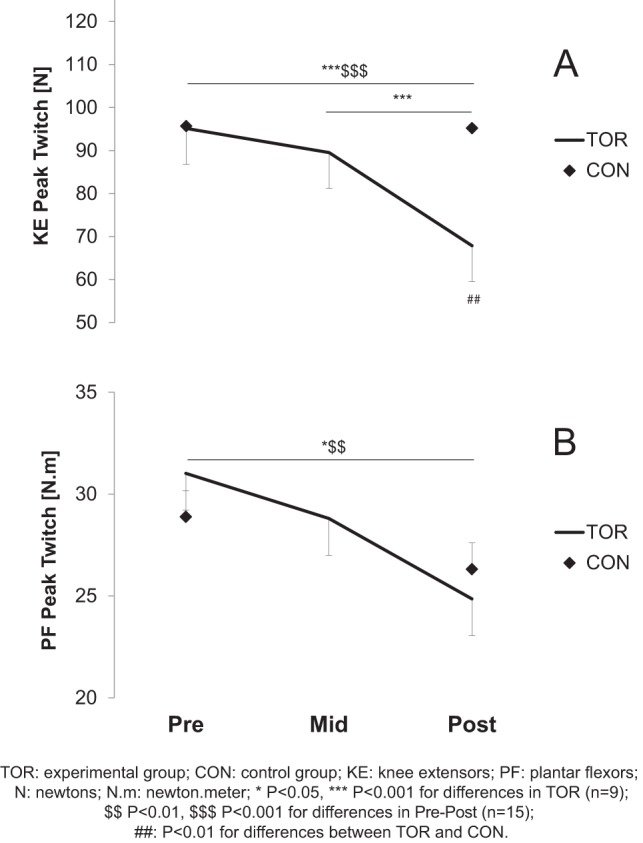
Amplitude of the potentiated peak twitch (Pt) prior to (Pre-), in the middle of (Mid-) and after (Post-) the race for knee extensors (KE, panel A) and plantar flexors (PF, panel B). A two-way repeated measure ANOVA was used complemented by a Bonferroni post-hoc test. Values are mean ± SE.

PS100 was significantly reduced for both muscle groups in TOR (−17.9±17.8% and −19.9±15.2% for KE and PF, respectively; P<0.01). No significant decrease was observed in CON (Figure 3). Peak twitch was significantly reduced for both KE and PF muscles in TOR (−33.6±12.3%, P<0.001 and −19.3±13.6%, P<0.01 for KE and PF, respectively), but no change was observed in CON (Figure 4). The PS10/100 ratio decreased significantly at Mid- for PF (−6.1±8.3%, P<0.01) but no change was reported for KE. The other peripheral fatigue markers are presented in Table 3.

**Table 3:** Values of potentiated peak twitch (N for knee extensors and Newton.meter: N·m for plantar flexors) and M-wave peak-to-peak amplitude (millivolts: mV) and peak-to-peak duration (ms) for the knee extensors (KE) and the plantar flexors (PF) prior to (Pre-), in the middle of (Mid-) and after (Post-) the race in runners (TOR, n = 9) and control group (CON, n = 8).

	KE						PF					
	TOR			CON			TOR			CON		
	Pre	Mid	Post	Pre	Mid	Post	Pre	Mid	Post	Pre	Mid	Post
**Potentiated Peak Twitch (N or N**·**m)**	95.1±22.2	89.5±23.4	67.9±21.3*	95.7±29.7	-	95.2±11.3**#**	31.1±6.5	28.8±9.4	24.9±5.5	28.9±6.8	-	26.3±9.4
**M-wave Peak-to-peak Amplitude (mV)**	15.1 ±8.8	14.1±5.9	12.5 ±5.8	17.5±7.3	-	14.9±7.8	9.5±2.7	9.6±4.3	12.8±6.6	11.6±2.1	-	9.9±3.6
**M-wave Peak-to-peak duration (ms)**	8.8 ±1.9	8.7 ±2.1	8.6 ±2.3	7.8±2.3	-	7.8 ±2.5	2.8±0.5	3.4±1.5	3.3±1.2	2.9±0.4	-	2.8±0.8

Values are given as mean ±SD. N = 8 and n = 9 for CON and TOR respectively. *: P<0.05 for differences with Pre; #: P<0.05 for differences between TOR and CON.

### Blood analysis

We found significant increases in the main inflammation and muscle damage markers in TOR. These results are presented in Table 4. Changes in Na^+^, K^+^, Ca^2+^, glucose, myoglobin and total protein are given in Tables 4 and 5. No significant correlation between the Pre- to Post- changes in neuromuscular parameters and changes in blood variables was found.

**Table 4:** Main blood markers of muscle damage and inflammation prior to (Pre-) and after (Post-) the race in runners (TOR, n = 9) and control group (CON, n = 6).

	Pre	Post
	Creatine Kinase (UI · L^−1^)	
**TOR**	112±33	3719±3045***
**CON**	122.5±41.1	147.7±32.6###
	**Lactate Dehydrogenase (UI · L^−1^)**	
**TOR**	340±51	1145±511***
**CON**	345±65	312±35###
	**C-Reactive Protein (mg · L^−1^)**	
**TOR**	0.31±0.32	13.11±7.51***
**CON**	1.05±1.04	0.65±0.61##
	**Creatinine (µmol · L^−1^)**	
**TOR**	0.94±0.12	0.95±0.15
**CON**	0.98±0.18	1.01±0.17
	**Myoglobin (µg · L^−1^)**	
**TOR**	25.6±5.5	449.3±338.2***
**CON**	26.1±8.4	32.3±16.4###
	**Total Protein (g · L^−1^)**	
**TOR**	72.7±3.9	64.9±4.6***
**CON**	72.4±2.9	73.1±3.4

Data are mean values ± SD. ***: P<0.001 for differences between Pre- and Post-. ##: P<0.01, ###: P<0.001 for differences between groups.

**Table 5:** Changes in Na^+^ K^+^, Ca^2+^ and glucose concentration prior to (Pre-) and after (Post-) the race in runners (TOR, n = 9) and control group (CON, n = 6).

	Pre	Post
	**Na^+^ (mmol · L^−1^)**	
**TOR**	141.1±1.9	138.3±3.4
**CON**	140.7±0.8	140.2±0.4
	**K^+^(mmol · L^−1^)**	
**TOR**	5.1±0.9	7.1±2.1*
**CON**	5.5±0.2	4.7±0.4##
	**Ca^2+^(mmol · L^−1^)**	
**TOR**	9.6±0.3	8.3±2.3***
**CON**	9.8±0.2	9.9±0.2###
	**Glucose (mmol · L^−1^)**	
**TOR**	4.8±0.7	4.3±0.8
**CON**	4.3±0.5	4.9±0.5**#

Values are given as mean ± SD. *: P<0.05, **: P<0.01, ***: P<0.001 for differences between Pre- and Post-. #: P<0.05, ##: P<0.01, ###: P<0.001 for differences between groups.

### Comparison with the UTMB study

**Figure 5:**
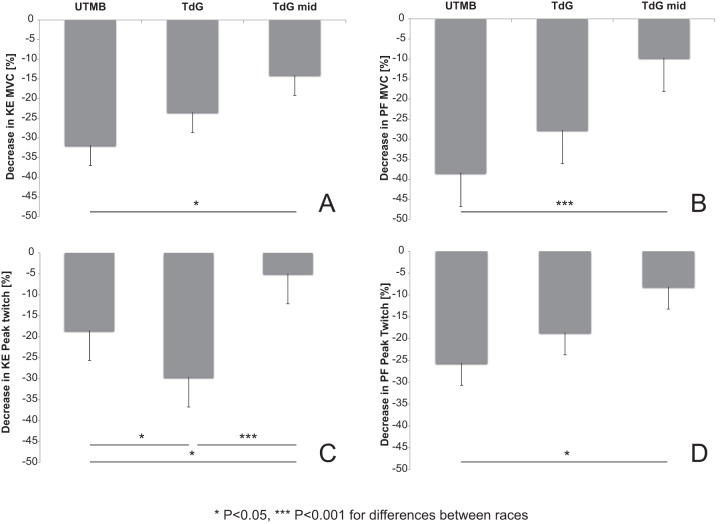
Decrease in MVC force for UTMB (Ultra-Trail du Mont-Blanc), the TdG (Tor des Geants) and TdG at mid-race (Mid) for the knee extensors (KE, panel A) and the plantar flexors (PF, panel B) and decrease in peak twitch for the UTMB, the TdG and TdG Mid for the knee extensors (panel C) and the plantar flexors (panel D). Unpaired t-tests were used. Data are given in changes from Pre- values.

We compared our main results to previous data collected in a previous study during UTMB, [3] (Figure 5). The anthropometrical values of the subjects were similar between TdG and UTMB. There was no significant difference between TdG at Mid- and UTMB in distance, elevation, running time or relative rank (Table 6). To compare the difference in speeds, we used a flat-equivalent speed calculated as:
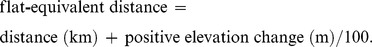



**Table 6:** Comparison of parameters concerning the different run (TdG: Tor des Geants; UTMB: Ultra Trail du Mont-Blanc) properties and subjects.

Parameters	TdG	TdG Pre-Mid	TdG Mid-Post	UTMB
**Equivalent flat distance (km)**	545	241	304	262
**Average equivalent-flat speed (km/h)**	5.5±2.8	6.2±2.1*	4.5±0.4*$	7.2±1.3*$†
**Speed decrease relative to UTMB (%)**	−23.1	−14.1*	−36.8*$	-
**D+ (m)**	24000	11500	12500	9600
**Relative rank (% field)**	34	-	-	38
**Time (h)**	107.5±15.4	39.1±5.9*	67.1±3.3*$	37.6±5.9*†
**Age (Yr)**	45.4±10.3	45.4±10.3	45.4±10.3	40.2±7.4
**Height (cm)**	173±5	173±5	173±5	178±7
**Weight (kg)**	69.4±6.1	69.4±6.1	69.4±6.1	73.4±6.4

Values are given in mean ± SD. Equivalent-flat distance was calculated as follows: (positive elevation (m)/100) + distance (km). *: P<0.05 different from TdG; $: P<0.05 different from TdG Pre-Mid; †: P<0.05 different from TdG Mid-Post.

### General fatigue and peripheral pain

**Figure 6:**
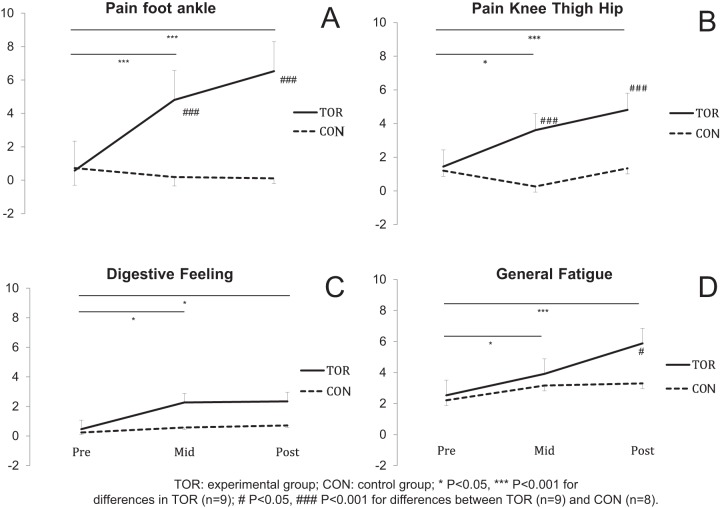
Visual analogue scale (VAS) for the pain-foot-ankle (panel A), the knee-thigh-hip (panel B), the digestive feeling (panel C) and the general fatigue (panel D) for runners (TOR) and control group (CON) prior to (Pre-), in the middle of (Mid-) and after (Post-) the race. A two-way repeated measure ANOVA was used complemented by a Bonferroni post-hoc test. Values are mean ± SE.

The changes in general fatigue, pain in foot-ankle and knee-thigh-hip and digestive feelings are displayed in Figure 6. The increase in these four variables was significant in TOR from Mid- whereas no changes were observed in CON.

No significant correlation was found between the changes in general fatigue or peripheral pain (measured with VAS) and the changes in neuromuscular parameters.

## Discussion

The purpose of the present study was to investigate neuromuscular and biological adjustments occurring during and after a single-stage MUM lasting between 4 and 7 days and inducing a large magnitude of sleep deprivation. The main results of the present study are that, compared to similar events of one-quarter to half the distance: (i) the neuromuscular function was generally less altered and (ii) muscle damage and inflammation markers (e.g. CK, LDH, C-RP) were much lower. This suggests that the pacing strategy (i.e. slow pace from the beginning of the race) and sleep deprivation that result in very low-intensity concentric/eccentric contractions preserve the neuromuscular function despite the apparent extreme difficulty of this event.

### Neuromuscular fatigue induced by the TdG

#### Central and peripheral alterations

At Post-MUM, MVC was significantly decreased in TOR but not in CON. When comparing the TdG with the UTMB [3] the decreases were lower for KE and tended to be lower for the PF (−24% vs. −32% in KE; −26% vs. −38% in PF, for TdG vs. UTMB, respectively). Surprisingly, over a MUM of longer distance and duration and greater elevation change, the magnitude of MVC force loss was lower than in shorter MUMs. These findings complement previous results presenting a MVC force loss – exercise duration relationship [6], [23]. In these previous studies, the authors described an increase in KE MVC force loss as running duration increases and then “a plateau for extreme durations.” This relationship was observed for durations between 2 and 37 h (i.e. less than 2 days). The present results highlight that for durations between 4 and 7 days, the loss in maximal force generating capacity may be even lower than the above-described “plateau.” This can be explained by the large deceleration throughout the MUM (Table 6) and consequently the relative low intensity during the second part of the race due to the accumulation of kilometers, elevation and sleep deprivation. Compared to shorter races, both the speed, especially at the end, and strength loss were lower.

It is interesting to compare the present results with a preceding study conducted on during the UTMB [3]. For a similar distance, running time and elevation, the decrease in MVC was two times greater in UTMB than Mid-TdG in KE (−31% *vs*. −13% for UTMB and Mid-TdG, respectively) and four times greater in PF (−40% *vs.* −10%). When compared to UTMB, the ∼15% lower running speed induced one-quarter to half the MVC force loss. These differences may be explained by the pacing strategy [24]. In the pacing strategy model, the athlete generates a conscious RPE managed by his brain, which assimilates all afferent information from each physiological system. The work output is then adjusted so that RPE does not increase disproportionately during the exercise, to avoid premature exercise termination. This pacing strategy is adopted by the runners in order to preserve themselves for the rest of the run; this can explain the lower decrease in MVC force observed Mid-TdG compared to UTMB.

The central component of fatigue after ultra-endurance exercise has been proposed as the main explanation for neuromuscular fatigue in KE [3], [4], [6], and it has been shown that this central drive alteration was not as high for PF as for KE after a 24–h treadmill run [25]. The present study partially confirmed these observations since the decrease in %CAR was significant in both muscle groups. These results could confirm the existence of a central mechanism already described in the UTMB [3] and whose purpose is “to reduce neural input to working muscles to limit fatigue and damage”[7], [26]. As expected, peripheral fatigue was also observed in this study, highlighted by the decreases in PS100 and peak twitch amplitude. Our results also suggest that muscle excitability was well-preserved [27] since no significant alteration was found in the M-wave properties. This confirms previous results obtained after various endurance exercises [23], [28]. Therefore, it rather seems that processes located beyond action potential propagation/transmission were impaired. The reduction in electrically evoked force can thus be attributed to the impairment of some steps of the excitation-contraction coupling mechanism and could involve (*i*) a reduced number of active cross-bridges due to a decreased release of Ca^2+^; (*ii*) a decreased sensitivity of the myofilaments to Ca^2+^; and/or (*iii*) a reduced force produced by each active cross-bridge [29]. Recent data seem to favor decreased Ca^2+^ release from the sarcoplasmic reticulum as an important contributor to fatigue after prolonged exercise and was explained by leaky ryanodine receptors, the Ca^2+^ release channels in the muscle [30]. This hypothesis is in accordance with the decrease in PS10/100 in the PF at Mid-, which revealed the presence of low-frequency fatigue (LFF) in this muscle as observed in the UTMB. In addition, it is interesting to note that for the same distance, time and elevation, the decrease in Pt was minimal (e.g. 5% and 8% at Mid- for the KE and PF respectively) and 3–4 fold greater in the UTMB (19% and 26%, respectively). This is in line with MVC loss described above and illustrates the conservative pacing strategy [7], [24], [31].

The reduction in speed, the sleep deprivation and the underlying respective feedback *vs.* feed-forward components of the pacing regulation are among the putative factors explaining this reduced strength loss observed in TdG, when compared to shorter distances. During the second part of the TdG (Mid-to-Post), the average flat-equivalent speed was 4.5 km·h^−1^ and was 37% lower than in UTMB (Table 6). Most of the participants were only walking during the final part of the TdG. Even the best athletes had a larger proportion of walking *vs.* running than in UTMB and thus less mechanical stress on the muscles [32]. This involved smaller forces on lower limb muscles resulting in a less eccentric work since downhill running leads to important eccentric contractions, especially for the KE [33], [34] knowing that muscle damage is greater with fast- than slow- velocity eccentric exercise [35]. In addition, the energetic cost of downhill walking is lower than for downhill running [36] and one cannot exclude that this might contribute to the pacing of the subjects. However, the influence of economy on MUM performance is unclear and is still being debated [37], [38].

We already mentioned that the reductions in MVC force and peak twitch, when compared to the UTMB, were likely due to a specific pacing strategy [24]. This is supported by the VAS “fatigue” and “pain” values that were all significantly lower at Mid- than at Post- (except “igestive feelings”). Of interest is that “general fatigue” at Mid- was not different between TOR and CON (Figure 6); however we cannot distinguish the influence of nociceptive feedback on this decrease in strength.

An important point in this study, which differentiates it from previous studies on ultra-endurance, is the severe sleep deprivation (Table 1). Although this possibility that sleep deprivation negatively affects voluntary strength has previously been proposed [10], it is difficult to attribute the loss of voluntary strength in TOR to the sleep deprivation since no significant strength loss was observed in CON constrained to the same amount of deprivation. The results of CON are in agreement with the rare studies examining the effects of sleep deprivation itself on neuromuscular function. On the contrary, several studies have illustrated an injurious effect of sleep deprivation on endurance performance [7], [39], [40]. Moreover, sleep deprivation one of the centrally acting performance factors presented in different models of performance regulation [7], [31] and can therefore influence pacing strategies. Indeed, a lack of sleep is known to have an influence on the RPE for a given exercise [39], [41]; i.e. the perceived exertion is higher with partial or total sleep deprivation. Thus, this factor can influence the pacing strategy by decreasing the speed in order to maintain a sufficient security reserve as described by Millet [7].

### Biological markers of fatigue

As expected, a 330-km/24000 m D+ (i.e. total positive elevation change) ultra-marathon led to a large increase in main blood markers of muscle damage and inflammation. As already observed in UTMB [3], there was a large variability among subjects for the biological parameters.

CK increased to a large extent in TOR whereas there was no change in CON. However, the post-race level of CK in TOR was much lower than after UTMB (3700 *vs.* 13600 UI.L^−1^ for TdG and UTMB, respectively). These results are also much lower than the values measured after the Western States Endurance Run (161 km and 5500 m D+/7000D-,[2]). It is well known that downhill running involves a large eccentric component, especially for the KE [33], [34] and leads to large increases in muscle damage markers, principally CK [42], [43]. Our relatively low CK values despite the extremely high downhill component of the TdG highlight the influence of running speed on the magnitude of muscle damage [43]. This relative muscle preservation is also illustrated by the increase in myoglobin concentration being much lower than after UTMB [5]. As discussed above, the final part of the TdG was walked by most of the participants mainly due to nociceptive feedback and sleep deprivation-induced fatigue. This is in line with CK values of ‘only’ 4500 UI.L^−1^ after a 1600-km “flat” ultra-marathon [44].

An interesting inflammation marker measured in our study was C-RP. This increased in TOR, showing a great inflammation process but this rise remained lower than the one observed in UTMB (46.8 *vs.* 13.1 mg·L^−1^ for UTMB and TdG, respectively), indicating less inflammation in TdG and in line with the lower muscle damage described above. Of interest is also that the increase in LDH concentration (+240%) was similar compared to the UTMB (+274%) [3], [5]. The decrease in total protein suggests an increase in plasma volume and these results are also in line with the UTMB data [5] and with other previous endurance exercises [45], [46]. This may confirm the inflammation process which had probably occurred in our case. Other mechanical parameters must be considered. The level of tension is recognized as a primary factor for exercise-induced muscle damage [47]. Since participants run faster in UTMB than on TdG, they consequently produced greater muscle forces during propulsive (concentric) actions. Stronger eccentric contractions were thus needed to absorb the potential energy during ground contact phase. More importantly, wider amplitudes are thus needed to perform these braking phases, especially for the knee and ankle joints [48]. The higher levels of tension associated with large movement amplitudes induce greater mechanical stress (lengthening) on muscle fibers [49], [50] and importantly affect the level of exercise-induced muscle damage [51]. These considerations could explain the present finding and are in line with the pacing strategy adopted and described above.

The purpose of this study was to assess the physiological impact of the world's most challenging mountainous ultra-marathon (TdG, 330 km; 24000 m D+). This competition is unique since it represents the greatest elevation change and distance ever performed over a single-stage mountainous ultra-marathon, inducing an amazing level of sleep deprivation. To our knowledge, the experimental design (inclusion of measurements at mid-race and comparison with a control group constrained to the same amount of sleep deprivation) is also unique and the first one allowing investigation of the pacing strategy and the responses to extreme fatigue induced by distance, elevation and sleep deprivation. We believe that ultra-endurance is an interesting model to better understand the pacing/coping strategies and adaptive responses of athletes facing extreme load and stress [1].

Over the TdG, an anticipatory pacing strategy was observed during the first part of the race. Then fatigue combined with a high level of sleep deprivation led to a large decrease in speed, particularly during the second part of the race, so that speed was relatively low over the whole course. This event induced lower inflammatory responses and less muscle damages than similar types of events of shorter duration, probably as a result of the very low concentric/eccentric contraction intensity due to the slow pace, showing that the amount of neuromuscular fatigue is not necessarily correlated to the difficulty of the event (duration and/or elevation). In addition, a control group allowed us to minimize the effects of sleep deprivation as the main factor of the neuromuscular fatigue in the runners.

In conclusion, beyond the influence of exercise duration on ultra-distance trails, the reduction in maximal force generating capacity seems to be related to other unidentified factors. Paradoxically, such an extreme MUM seems to induce relative muscle preservation. Further studies focusing on injuries and pain are required to complete the understanding of the physiological impact of such non-standard exertions.
